# Perfusate Composition and Duration of *Ex-Vivo* Normothermic Perfusion in Kidney Transplantation: A Systematic Review

**DOI:** 10.3389/ti.2022.10236

**Published:** 2022-05-11

**Authors:** Amir Fard, Robert Pearson, Rashida Lathan, Patrick B. Mark, Marc J. Clancy

**Affiliations:** ^1^ Institute of Cardiovascular and Molecular Sciences, Glasgow University, Glasgow, United Kingdom; ^2^ Queen Elizabeth University Hospital, Glasgow, United Kingdom

**Keywords:** review, kidney, perfusion, normothermic, perfusate

## Abstract

*Ex-vivo* normothermic perfusion (EVNP) is an emerging strategy in kidney preservation that enables resuscitation and viability assessment under pseudo-physiological conditions prior to transplantation. The optimal perfusate composition and duration, however, remain undefined. A systematic literature search (Embase; Medline; Scopus; and BIOSIS Previews) was conducted. We identified 1,811 unique articles dating from January 1956 to July 2021, from which 24 studies were deemed eligible for qualitative analysis. The perfusate commonly used in clinical practice consisted of leukocyte-depleted, packed red blood cells suspended in Ringer’s lactate solution with Mannitol, dexamethasone, heparin, sodium bicarbonate and a specific nutrient solution supplemented with insulin, glucose, multivitamins and vasodilators. There is increasing support in preclinical studies for non-blood cell-based perfusates, including Steen solution, synthetic haem-based oxygen carriers and acellular perfusates with supraphysiological carbogen mixtures that support adequate oxygenation whilst also enabling gradual rewarming. Extended durations of perfusion (up to 24 h) were also feasible in animal models. Direct comparison between studies was not possible due to study heterogeneity. Current evidence demonstrates safety with the aforementioned widely used protocol, however, extracellular base solutions with adequate oxygenation, supplemented with nutrient and metabolic substrates, show promise by providing a suitable environment for prolonged preservation and resuscitation.

**Systematic Review Registration:** https://www.crd.york.ac.uk/prospero/display_record.php?ID=CRD42021231381, identifier PROSPERO 2021 CRD42021231381

## Graphical Abstract

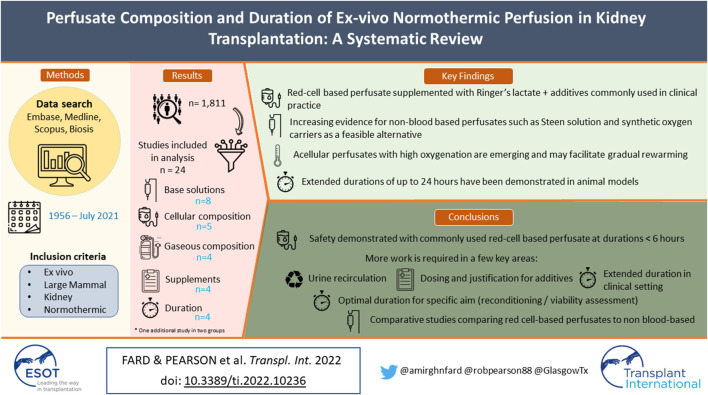

## Introduction

Kidney transplantation is the gold standard treatment for end stage renal disease. The mainstay of organ preservation has traditionally focused on reducing metabolism by utilising hypothermic conditions with static cold storage (SCS) or, more recently, hypothermic machine perfusion (HMP) (1). The continued donor organ shortage has necessitated increased use of kidneys from donation after circulatory death (DCD) and “extended criteria” donor (ECD), (2) which are more susceptible to the effects of ischaemia reperfusion injury (IRI). IRI is multifactorial process that results in an increase in reactive oxygen species (ROS) and inflammatory mediators which stimulate vascular permeability leading to oedema and vascular endothelial damage (3–5). Furthermore, the effects of IRI are associated with higher rates of acute rejection, delayed graft function (DGF), and reduced long-term allograft survival (4). Preservation techniques to mitigate against the effects of IRI are therefore of increasing importance.

One emerging strategy is *ex-vivo* normothermic perfusion (EVNP). This involves rewarming the graft to normothermic conditions (37°C) with a perfusate that replicates the pseudo-physiological environment. Thus, facilitating the restoration of energetic substrates (e.g., ATP), metabolism and repair processes, whilst also facilitating graft viability assessment. Recently, the safety and feasibility of EVNP has been established in human clinical studies (6,7). Although unlikely to entirely counteract the process of IRI, EVNP has the potential to mitigate these deleterious effects during the period of perfusion (6).

The ideal perfusion characteristics including perfusate composition and duration remain undefined. Common clinical protocols employ a nutrient-enriched, red blood cell (RBC)-based perfusate to deliver nutrients and oxygen during 1-hour of perfusion (6,8). In addition to prolonging the duration of EVNP, variations in composition, such as synthetic and acellular preparations with varying base media, have been proposed in preclinical studies and established in liver and lung clinical protocols. However, major deviations have yet to be clinically implemented in kidneys, and limited evidence exists for the impact of different perfusion characteristics. The aim of this review was to summarise the evidence for the roles of perfusate constituents and the effects of different perfusion durations in optimising clinically relevant outcomes in the context of renal EVNP.

## Materials and Methods

### Data Sources and Search Strategy

For this systematic review, we followed the methods proposed by the Preferred Reporting Items for Systematic Reviews and Meta-Analyses (PRISMA) statement, (9) and the Cochrane Handbook for Systematic Reviews of Interventions. This review was registered with PROSPERO (CRD42021231381) (10).

A limited search of the literature was conducted to identify keywords, followed by an extensive literature search on the following databases: Embase (Ovid) 1947-Present; Ovid Medline® without Revision; Scopus; and BIOSIS Previews. The keywords used to identify relevant studies included normothermic perfusion and evnp and kidney; a comprehensive description of the search strategy can be found in Supplementary Appendix S1. Results were imported into Rayyan QCRI web application, where duplicate articles were removed, then two main reviewers independently and blindly screened the titles and abstracts based on predefined eligibility criteria. Thereafter, selected studies were read in full. Bibliographies of the selected articles were screened to identify landmark trials.

### Eligibility Criteria

The eligibility criteria were agreed based on the study objectives and specific research question: what are the roles of various perfusate constituents, and what are the effects of different durations of perfusion on clinically relevant outcomes in renal EVNP?

Eligible studies included preclinical and clinical, published and abstract publications from any year and any region, where English translations were available. Studies that were unpublished and those concerning *in vivo* perfusion methods, non-large mammal studies, non-kidney studies, assessment of perfusate biomarkers, and therapeutic interventions were excluded. Articles relating to sub-normothermic perfusion methods were only included where specific rationale for perfusate composition was discussed.

### Data Extraction and Analysis

The most recently dated studies were read in full first to identify up-to-date knowledge and previous related studies. Study characteristics, including name, year, design, subjects, objectives, perfusate composition, perfusion duration, main outcome measures and key findings were recorded.

## Results

**FIGURE 1:**
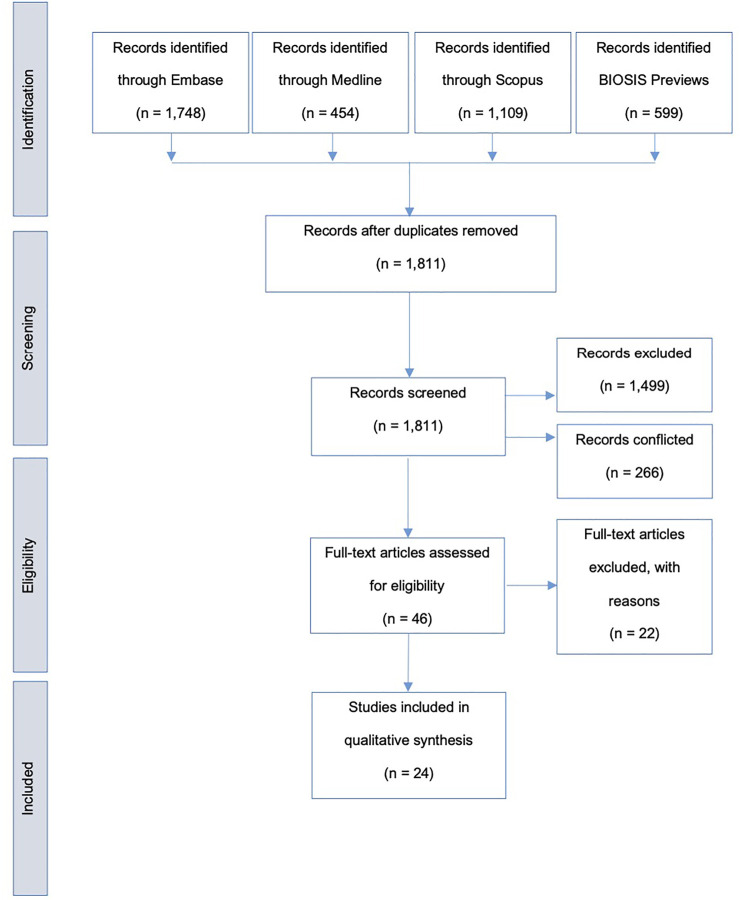
Search strategy flow diagram; adapted from the preferred reporting items for systematic reviews and meta-analyses (PRISMA) flow diagram (9).

The search identified 3,910 articles, 2099 of which were duplicates, giving 1,811 unique articles, dating from January 1956 to July 2021. Following blinded screening by two independent reviewers, 1,499 articles were deemed ineligible, with 266 decisions conflicted. A third reviewer was used to address conflicts. Of the articles selected, 46 met the eligibility criteria. Full-text assessment reasoned a further 22 articles ineligible for qualitative analysis. Only studies utilizing human or large mammal tissue were included. Figure 1 illustrates the search process in full.

Included studies were grouped according to common themes: Whole perfusates and base solutions (*n* = 8); cellular composition (*n* = 5); gaseous composition (*n* = 4); supplementary composition (*n* = 4); and perfusion duration (*n* = 4), with one study applicable to both whole perfusate and base solutions, and perfusion duration. Studies comprised 5 clinical studies on human patients and 19 preclinical studies. Key findings were recorded and summarised in Table 1 for perfusate composition and Table 2 for perfusion duration.

**TABLE 1:** Summary characteristics of perfusate composition studies qualitatively assessed.

Theme	Study	Design	Subject model	Objectives	Main outcome measures	Key findings
Whole perfusates and base solutions	Hosgood SA et al; 2011(6)	Published; clinical case report	Human patient (*n* = 1)	First EVNP in human renal transplantation	Renal hemodynamics (renal blood flow, resistance, urine output); post-transplant serum creatinine; graft function	EVNP with plasma-free red cell-based perfusate is feasible
	Nicholson ML et al; 2013(7)	Published; clinical study	Human patients (*n* = 18)	First clinical series EVNP in human renal transplantation	Graft primary nonfunction; delayed graft function (DGF)—need for dialysis; graft failure—need for nephrectomy or RRT	DGF was 5.6% in EVNP group vs. 36.2% in SCS group (*p* = 0.014); no difference of graft or patient survival at 12 months
	Hosgood SA et al; 2016(8)	Published; clinical case report	Human patients (*n* = 2)	First clinical EVNP transplantation of DCD kidneys deemed untranslatable	Graft hemodynamics; posttransplant graft function; serum creatinine	Serum creatinine at 3 months was 1.2 mg/dl and 1.62 mg/dl in the recipient of the left and right kidney—EVNP rescued kidneys previously deemed unsuitable for transplantation
	Hosgood SA et al; 2017(11)	Published; Protocol of clinical trial	Human patients (*n* = 400 for recruitment)	1-hour renal EVNP in kidneys from DCD donors versus SCS	Primary: DGF (need for dialysis in first 7-day); Secondary: renal function, hospital stay, graft & patient survival at 1 year; acute rejection; blood chemistry biomarkers	Study suspended during COVID-19 pandemic and preliminary results not yet available
	Horiuchi T et al; 2009(14)	Published; preclinical	Canine kidneys	Pyridoxalated hemoglobin-polyoxyethylene (Php) addition to UW solution for normothermic preservation	Oxygen consumption; histopathological assessment	Php added to UW during 12-hour normothermic preservation increased oxygen consumption, reduced damage of tubular epithelium and edematous degeneration compared to UW alone
	Kaths JM et al; 2015(35)	Published; preclinical	Beating-heart porcine kidneys (*n* = 6)	EVNP using erythrocyte-based Steen solution diluted with LR perfusate	Renal hemodynamics; blood gas analysis; histopathological assessment	10-hour DCD porcine perfusion using erythrocyte-based Steen solution diluted with ringer’s lactate demonstrated stable hemodynamics, active renal metabolism and minimal renal injury
	Urcuyo D et al; 2017(12)	Published; preclinical	Porcine kidneys (*n* = 15)	Whole-blood at normothermia, whole-blood with Steen solution at normothermia, and acellular Steen solution at sub-normothermia, on prolonged preservation	Primary: Hemodynamic stability and histological damage Secondary endpoints: Urine production, perfusate potassium and arterial pH	Acellular Steen solution at 21°C supported low and stable vascular resistance with adequate histological preservation during 24-hour perfusion; whole blood diluted with Steen solution at normothermia was successful but resulted in acidosis and necrosis. Whole blood alone at normothermia was unsuccessful beyond 5-hours
	Horn CV et al; 2021(15)	Published; preclinical	Porcine Kidneys (n = 12)	New preservation solution Custodiol-MP for *ex vivo* reconditioning of kidney grafts compared to Belzer MPS solution	Primary: renal haemodynamics Secondary: Molecular markers of renal injury and histology	No statistically significant difference in outcomes between Custodiol-MP and Belzer MPS solutions. Custodiol-MP was safe and applicable for short-term kidney perfusion
	Pool MBF et al; 2021(36)	Published; preclinical	Porcine Kidneys (*n* = 20)	Comparison of four different perfusate solutions	Perfusion parameters, Urine and perfusate analysis, Markers of renal injury, Histology	All four perfusates were feasible but with differences in outcome measures. Individual influence of perfusate components remain unclear
Cellular Composition	Harper S et al; 2006(16)	Published; Preclinical	Porcine kidneys (*n* = 12)	Leukocyte-depleted blood versus whole blood-based perfusates	Serum creatinine, urine output, renal blood flow, oxygen consumption, acid-base homeostasis, histological features	Leukocyte-depleted blood significantly improved post-ischemia renal function; lower serum creatinine, higher creatinine clearance and urine output (*p* = 0.002 for all)
	Aburawi MM et al; 2019(17)	Published: Preclinical	Discarded human kidneys (*n* = 14)	Hemoglobin-based oxygen carriers (HBOC) versus pack red blood cell-based perfusates	Renal artery resistance, oxygen extraction, metabolic activity, energy stores and histological features	Lactic acid levels in kidneys pRBC group was higher than HBOC group (*p* = 0.007); other outcomes were similar
	Minor T et al; 2019(13)	Published: preclinical	DCD Porcine kidneys (*n* = 12)	RBC-based perfusate versus acellular perfusate versus control during controlled rewarming	Renal hemodynamics and histological assessment	Controlled organ rewarming is superior to immediate rewarming in terms of creatinine clearance, sodium excretion, oxygen extraction, urinary protein loss and innate immune activation; inclusion of RBC added no benefit
	Minor T et al; 2019(18)	Published: clinical case report	Human Patient (*n* = 1)	First controlled rewarming with an acellular Steen perfusate in human renal transplantation	Post-transplant immediate graft function; serum creatinine; urine output; patient outcomes	Postoperative course was event-free, and patient was discharged after 16 days with a serum creatinine of 143 μmol/L; Acellular controlled oxygenated rewarming was successful
Gaseous Composition	Adams TD et al; 2019(19)	Published; preclinical	Porcine kidneys (*n* = 43)	Effects of reducing perfusate oxygenation on renal function and oxygen kinetics during EVNP and reperfusion	Renal function and hemodynamics; blood gas analysis; biomarkers of renal injury (NGAL)	Reducing partial pressure of oxygen significantly reduced oxygen extraction during EVNP (*p* = 0.037) however showed no significant difference in urine output, sodium excretion, creatinine clearance or NGAL during reperfusion
	Maasseen H et al; 2019(21)	Published: preclinical	Porcine kidneys (*n* = 10)	Hydrogen sulphide versus control	Renal function and hemodynamics; oxygen kinetics; histopathological assessment; metabolic activity	Hydrogen sulphide significantly reduce oxygen consumption, by 61%, (*p* = 0.047) without directly affecting tissue ATP levels. Renal function was unchanged
	Bagul A et al; 2008(20)	Published; preclinical	Porcine kidneys (*n* = 4)	Effect of carbon monoxide	Renal function and hemodynamics	Carbon monoxide improved renal blood flow (*p* = 0.002), creatinine clearance (*p* = 0.006), and urine output (*p* = 0.01). Higher concentrations had negative effects
	Smith SF et al; 2017(22)	Published: preclinical	Porcine kidneys (*n* = 18)	70% argon versus 70% nitrogen versus 95% O_2_ 5% CO_2_ during EVNP	Renal function and hemodynamics; inflammatory mediators and histopathological assessment	Argon did not mediate any significant effects during EVNP nor reperfusion during functional parameters, inflammatory mediators or histological changes
Supplementary Composition	Bleilevens C et al; 2019(23)	Published; preclinical	Porcine kidneys (*n* = 10)	Vitamin C versus placebo in an *in vitro* ischemia-reperfusion porcine kidney EVNP model	Perfusate analysis (blood gas, serum chemistry, oxidative stress markers); renal hemodynamics; histological analysis	Vitamin C significantly increased antioxidant capacity and hemoglobin concentrations (*p* = 0.02), reduced oxidative stress (*p* = 0.002) however did not improve creatinine clearance, fractional sodium excretion or renal histology
	Hosgood SA et al; 2017(25)	Published; preclinical	Porcine kidneys (*n* = 10)	Effect of a CytoSorb heme-adsorber in an isolated kidney perfusion system	Tissue and blood markers of inflammation and renal function	In the cytosorb group, interleukin-6/8, prostaglandin E2 and thromboxane were significantly lower during reperfusion (*p* = 0.023, *p* = 0.0001 and *p* = 0.005 respectively) and renal blood flow was significantly higher (*p* = 0.005); creatinine clearance was not significantly difference (*p* = 0.109)
	Brasile L et al; 2003(26)	Published: preclinical	Canine kidneys (*n* = 32)	Feasibility of cobalt protoporphyrin (CoPP) on heme-oxygenase (HO-1) expression during acellular warm perfusion	HO-1 activity; Renal hemodynamics	Induction of HO-1 during warm acellular perfusion by CoPP is feasible within clinical timeframe
	Yang B et al; 2011(24)	Published: preclinical	Porcine kidneys (*n* = 6)	Impact of EPO addition to 2-hour RBC-based EVNP	Renal hemodynamics; immunohistochemistry, histopathological assessment	EPO in EVNP significantly facilitated inflammation clearance and improved and urine output

EVNP, *Ex-vivo* normothermic perfusion; SCS, Static cold storage; DGF, Delayed graft function; UW, University of Wisconsin solution; LR, lactate Ringer’s solution; Php, Pyridoxalated hemoglobin-polyoxyethylene; DCD, Donation after circulatory death; ECD, Expanded criteria donor; HBOC, hemoglobin-based oxygen carriers; pRBC, Pack red blood cells; CoPP, Cobalt Protoporphyrin; HO-1, Heme-oxygenase 1; EPO, Erythropoietin; IRI, ischemia-reperfusion injury.

**TABLE 2:** Summary characteristics of kidney perfusion duration studies qualitatively assessed.

Study	Design	Subject model	Objectives	Duration groups	Main outcome measures	Key findings
Kaths JM et al; 2016(52)	Published: preclinical	SCD Porcine kidneys (*n* = 10)	Safety and feasibility of 8-hour EVNP versus SCS	(A) SCS (8 h)	Perfusate injury markers (AST, LDH); Renal function (serum creatinine, 24-hour creatinine clearance); Histological assessment	Continuous EVNP is feasible and safe in good quality beating-heart donor kidney grafts
				(B) EVNP (8 h)		
Kaths JM et al; 2017(28)	Published: preclinical	DCD Porcine kidneys (*n* = 20)	Brief EVNP following SCS versus prolonged, continuous EVNP in DCD porcine kidney autotransplantation	(A) 16 h SCS	Perfusate injury markers (AST, LDH); Renal function (serum creatinine, 24-hour creatinine clearance), Histological assessment	Prolonged EVNP significantly decreased serum creatinine, LDH, and apoptotic cells following DCD kidney transplantation compared to SCS or short EVNP after SCS.
				(B) 15 h SCS + 1 h EVNP		
				(C) 8 h SCS + 8 h EVNP		
				(D) 16 h EVNP		
Kaths JM et al; 2017(27)	Published: preclinical	DCD Porcine kidneys (*n* = 35)	Brief versus intermediate versus prolonged EVNP following 8-hours SCS in DCD porcine kidney autotransplantation	(A) 8 h SCS	Renal function and hemodynamics; Histological assessments 8 days post-transplantation	Intermediate and prolonged EVNP were significantly superior to brief EVNP following SCS. Brief EVNP resulted in a higher serum creatinine compared to SCS alone
				(B) 8 h SCS + 1 h EVNP		
				(C) 8 h SCS + 8 h EVNP		
				(D) 8 h SCS + 16 h EVNP		
Urcuyo D et al; 2017(12)	Published: preclinical	DCD Porcine kidneys (*n* = 15)	Whole-blood at normothermia versus whole-blood with Steen solution at normothermia, and acellular Steen solution at sub-normothermia, on prolonged preservation	(A) 24 h EVNP with whole blood	Primary: Hemodynamic stability and histological damage	Acellular Steen solution at 21°C supported low and stable vascular resistance with adequate histological preservation during 24-hour perfusion; whole blood diluted with Steen solution at normothermia was successful however resulted in acidosis and necrosis. Whole blood alone at normothermia was unsuccessful beyond 5-hour
				(B) 24 h EVNP with whole blood + Steen solution	Secondary endpoints: Urine production, perfusate potassium and arterial pH	
				(C) 24 h sub-normothermic preservation with acellular Steen solution		

SCD, Standard criteria donor; SCS, Static cold storage; EVNP, *Ex-vivo* normothermic perfusion; AST, Aspartate transaminase; LDH, Lactate dehydrogenase; DCD, Donation after cardiac death.

Qualitative analysis found the perfusate commonly implemented in clinical renal EVNP consisted of Ringer’s lactate, O-negative packed red blood cells (pRBC), Mannitol 10%, dexamethasone 8 mg, heparin, Sodium bicarbonate 8.4% as the main components, and a specific nutrient solution with insulin, multivitamins, prostacyclin 0.5 mg and glucose 5% as supplementary components, for a perfusion duration of 1-hour following SCS, pioneered by Nicholson et al. in Cambridge (7).

Preservation solutions are broadly categorised into intracellular and extracellular solutions, pertaining to whether the potassium and sodium concentrations mirror that of the intra- or extra-cellular milieu. Regarding the base solutions used for perfusate at normothermia, extracellular electrolyte compositions such as Ringer’s lactate have demonstrated safety and feasibility when implemented in human clinical studies; although lacking robust data, the perfusion pressure maintained in human trials thus far ranges from 65 to 75 mmHg (6,8,11). In addition, Steen-based solutions, with or without RBCs, have been shown to support prolonged perfusion up to 24-hour of EVNP of DCD porcine kidneys (12,13). One study on isolated canine kidneys showed that addition of pyridoxalated haemoglobin-polyoxyethylene (Php) to UW solution enhanced oxygen consumption and reduced oedematous damage of tubular epithelium during 12-hour normothermic preservation, however, no studies have yet translated this into clinical models (14). Custodiol-MP solution was safe and feasible for short-term perfusion of porcine kidneys, and non-inferior to clinically established Belzer MPS solution. Head-to-head comparison of four different perfusates showed feasibility in all settings during 7-hour EVNP of porcine DCD kidneys, but with substantial differences in perfusion and injury parameters (15). In this instance, the influence of individual perfusate components remains unclear.

For cellular composition, leukocyte-depleted blood significantly improved post-ischaemia renal function by measure of serum creatinine and urine output (*p* = 0.002) in porcine kidneys (16). Perfusates utilising synthetic haemoglobin-based oxygen carriers (HBOCs) were found to be non-inferior to whole blood perfusates with regard to histological injury, vascular resistance, oxygen consumption and tissue ATP, and exhibited significantly lower lactic acid levels (*p* = 0.007) during perfusion.(17) Controlled oxygenated rewarming without any oxygen carriers resulted in successful transplantation with good immediate renal function, in a recent human clinical case study.(18).

Evidence for gaseous composition supported 95% oxygen (O_2_), 5% carbon dioxide (CO_2_) mixtures. Reducing oxygen levels to normoxia significantly reduced oxygen consumption during EVNP (*p* = 0.037), however showed no difference in urine output, sodium excretion, creatinine clearance or markers of injury during reperfusion (19). The addition of carbon monoxide (CO) improved renal blood flow (*p* = 0.002), creatinine clearance (*p* = 0.006), and urine output (*p* = 0.01), however higher concentrations had negative effects (20). Despite being commonly known for its toxicity, the infusion of hydrogen sulfide (H_2_S) to the perfusate was found to induce a hypometabolic state, significantly reducing oxygen consumption by 61%, (*p* = 0.047) without directly impacting tissue ATP levels, and renal function was unchanged (21). Argon did not mediate any significant effects during EVNP or during reperfusion (22).

Evidence for supplementary additives was limited. While vitamin C significantly increased antioxidant capacity, haemoglobin concentrations (*p* = 0.02), and reduced oxidative stress (*p* = 0.002); it was not shown to improve creatinine clearance, fractional sodium excretion or histological markers of renal tubular injury (23). In a porcine model EPO was found to be anti-inflammatory and anti-apoptotic, demonstrating improved urine output with the mechanism attributed to caspase-3 and IL-1β (24). Reduction of inflammatory mediators was also demonstrated to be achieved by filtration via CytoSorb haemadsorption, which significantly reduced interleukin (IL)-6/8, prostaglandin E2 and thromboxane during reperfusion (*p* = 0.023, *p* = 0.0001 and *p* = 0.005 respectively), and increased renal blood flow (*p* = 0.005) without significantly altering creatinine clearance (*p* = 0.109).(25) In addition, induction of haem-oxygenase-1 (HO-1) was demonstrated in canine kidneys however evidence for clinical impact is yet to be elucidated (26). Commonly used protocol for clinical use and prominent variations in perfusate constituents, along with their roles, are summarized in Tables 3, 4, respectively.

**TABLE 3:** Perfusate composition commonly used for clinical renal ex-vivo normothermic perfusion; adapted from the nicholson protocol (6, 7, 11).

	Constituent	Volume
Components	Ringer’s lactate solution	300–400 ml
	O-negative packed red blood cells (leukocyte depleted) from blood bank	1 Unit
	Mannitol 10%	25 ml
	Dexamethasone 8 mg	Direct to circuit
	Sodium Bicarbonate 8.4%	25 ml
	Heparin 1,000 iu/ml	2 ml
Supplement	Nutrient solution (Nutriflex or Synthamin)	20 ml/h infusion
	Sodium Bicarbonate 8.4%	20 ml/h infusion
	Insulin 100 iu	20 ml/h infusion
	Multivitamins (Cernevit)	20 ml/h infusion
	Prostacyclin 0.5 mg	5 ml/h infusion
	Glucose 5%	5 ml/h infusion
	Ringer’s lactate solution	Replace urine output ml for ml

**TABLE 4:** Clinical Perfusate Constituent Options summary; Adapted from of Qualitative Analysis of Studies.

	Component role	Clinical constituent options
Base Solution	Fluid and electrolyte balance	Ringer’s Lactate
		Steen solution
	Elevation of osmolality	Mannitol 10%
	pH Buffer	Sodium Bicarbonate 8.4%
	Calcium Buffer	Calcium Gluconate 10%
	Immune suppression	Dexamethasone 8 mg
	Anticoagulation	Heparin 1,000 iu/ml
Cells	Oxygenation	Plasma free, leukocyte-depleted packed Red Blood Cells (1 unit)
		Synthetic Heme-based oxygen carriers
		Acellular with no oxygen carrier
Gases	Oxygenation	Carbogen gas mixture (95% O_2,_ 5% CO_2_)
	Hypo-metabolite	Hydrogen sulphide (H_2_S)
	Vasodilation	Carbon Monoxide (CO)
Supplementary Component	Nutrition	Nutrient solution (Nutriflex)
		Synthamin 17 (500 ml)
	pH Buffer	Sodium Bicarbonate 8.4% (25 ml)
	Energetic & metabolic substrates substrate	Insulin 100 iu
		Glucose 5%
	Nutrition solution	Multivitamins (Cernevit) (1 vial)
	Vasodilation	Prostacyclin 0.5 mg
		Verapamil 0.25 mg/h
	Replace fluid lost in urine output	Ringer’s Lactate (ml for ml)
	Inflammatory suppression	Heme-oxygenase-1 (HO-1)

Continuous EVNP, with and without complete exclusion of SCS, was feasible and superior to brief EVNP (27,28). 8-hour and 16-hour durations showed significantly lower post-transplant serum creatinine compared to 1-hour EVNP (*p* = 0.027), with no significant difference between the former (28). Acellular Steen solution at 21°C supported low and stable vascular resistance with adequate histological preservation during 24-hour perfusion, compared to whole blood alone at normothermia, which was unsuccessful beyond 5-hour (12).

## Discussion

In this systematic review, the most recent evidence for roles of various EVNP perfusate constituents and durations in optimising clinically relevant outcomes of kidney transplantation were reviewed and summarised.

### Fundamentals of Perfusate Composition and Current Clinical Practice

Preservation of organs at normothermia requires a physiological milieu with adequate oxygen, nutrition, and metabolic substrates to replace depleted energy resources. Furthermore, it is necessary that the solution stabilises electrolyte balance and cell fluid content to reduce oedema and reduce free radical peroxide scavengers to diminish oxidative injury (29). Accordingly, the protocol most commonly utilised in clinical practice, (6,7) comprises a nutrient enriched, red cell-based solution, with physiological buffers and added supplementary constituents such as vitamins, insulin, glucose and vasodilators (14,30,31).

### Base Solutions

Early evidence has shown that, under normothermic conditions, colloid solutions with high-sodium, low-potassium compositions like that of extracellular fluid, such as Ringer’s lactate, are superior to UW, which has a low-sodium, high-potassium composition like that of intracellular fluid, by reducing temperature-dependent oedema during IRI (31). This is consistent with evidence that clinical implementation of renal EVNP using Ringer’s lactate solution is feasible (6,7,11). Further work is required to elucidate optimal mean arterial pressure (65–75 mmHg non-pulsatile is most commonly reported as target pressure), particularly in the setting of high resistance kidneys where some groups describe increasing pressure to 100 mmHg to promote perfusate flow (11).

Steen solution is alternative plasma-like solution that was initially utilised for EVNP of the lungs in the Toronto Protocol (32), and has since been developed in liver EVNP (33,34). It contains dextran and a high albumin concentration that provides oncotic force to drive water out of swollen endothelial cells, helping sustain high perfusion flow rates (12). For use with EVNP, it can remain acellular or be supplemented with RBCs. Recent studies using similar protocols in kidneys have shown that Steen solution-based perfusates can support low and stable vascular resistance during prolonged perfusion, superior to red cell-based perfusates (12). Gaining popularity is Ringer’s lactate diluted with Steen solution, which has been successfully implemented in porcine kidneys for up to 10 h of EVNP, both with RBCs (35) and without (12). Further research is required to compare these different base solutions at normothermia, and to explore the potentially protective effects of Php.

Another emerging product is Custodiol-MP solution, which is reported to have antioxidant properties, specifically designed for aerobic or oxygenated machine perfusion. Compared to Belzer MPS, Custodiol-MP was deemed safe for short-term kidney perfusion, and while there were no statistically significant differences in renal hemodynamic outcomes, it remains an attractive solution which may benefit from testing in further models, as it allows flexible addition of colloids, specific to the requirements of each organ, potentially enabling wider clinical application (15).

Few studies to date have conducted head-to-head comparisons of perfusates for EVNP. A recent publication from Pool et al., however, compared four different perfusates during 7-hour EVNP of porcine kidney in a DCD model (36). While all four perfusates demonstrated feasibility, there were apparent differences between electrolyte levels, renal function parameters, and injury markers in the four groups. Perfusate 1, consisting of RBCs in Williams’ Medium E-based solution, and Perfusate 2, consisting of RBCs, albumin and balanced electrolyte solution, were similar in terms of EVNP flow patterns, whereas Perfusate 3, consisting of RBCs with clinically established solution used by Hosgood et al., (7) and Perfusate 4, consisting of RBCs and a 0.9% sodium chloride-based medium (successfully used in porcine autotransplantation, (37) showed lower but more stable flow rates. This may be explained by a lack of vasodilator use in Perfusates 1 and 2. Notably, Perfusate 2 resulted in significantly lower levels of injury marker N-acetyl-β-D glucosaminidase compared to Perfusate 3 and 4, and where Perfusate 3 had the highest levels, indicating greatest tubular damage. Ultimately, this study highlighted the significant influence of different perfusate compositions on EVNP outcomes, and the importance of a harmonious protocol to enable consistent interpretation of EVNP data. The need for further comparative studies to assess these perfusate protocols is self-evident in order to further this perfusion technology.

### Cellular Composition

Most preclinical studies to date have used red cell-based perfusates; however, it is important to note that whole blood is a finite resource, particularly given that type O packed erythrocytes is most commonly used. Furthermore, the blood may contain antibodies, clotting factors, activated leukocytes and thrombocytes which potentially exacerbate IRI through generation of inflammatory mediators and activation of complement cascade (16). Accordingly, plasma-free and leukocyte-depleted perfusates have been well-established in both preclinical and clinical studies (7, 8). However, there is limited data on whether or not plasma-based perfusates, or the use leucocyte depletion filters, have a role in wider clinical use.

Nevertheless, adequate oxygenation remains a vital prerequisite, which can be delivered by several means: RBCs, synthetic HBOCs or simple diffused oxygen by carbogen gas mixtures. While RBC-based perfusates are proven, they are limited by poor availability, high cost and short-shelf life, with potentially increased risk of infection transmission and haemolysis (17). HBOCs are more accessible with reduced infection and haemolysis risks (17). Recently, preclinical studies on discarded human kidneys have demonstrated that HBOCs are non-inferior to pRBCs in terms of renal hemodynamics and histological damage (17), suggesting that HBOCs may indeed offer a logistically more convenient alternative to pRBC in EVNP of human kidneys. Further studies, however, demonstrating improved clinical outcomes in appropriate transplant models are required.

Acellular perfusates, without any haem-based oxygen carriers, may offer a unique benefit as they better enable gradual rewarming of the organ to normothermia. At present, EVNP is performed at the receiving site after a period of SCS transport from the donor hospital. This abrupt restoration of normothermia and rise in metabolic turnover has been implicated as a secondary cause of IRI (5). This is thought to be due to disrupted cellular homeostasis at the mitochondrial level (5) and to RBCs losing their deformability in cold, leading to impaired microcirculation and tissue oxygenation, and can be mitigated by gently rewarming the organ from SCS using an acellular perfusate (13). It has been demonstrated (data presented at ATC 2019) that EVNP may be feasible without haem-based oxygen carriers for up to 6 h in discarded human kidneys (38). In this instance, the perfusate, with 95% O_2_, 5% CO_2_, sustained stable renal haemodynamics and restored tissue ATP levels similar to concentrations in a red cell-based perfusate. Acellular EVNP of porcine kidneys has also been shown to fully saturated venous haemoglobin when the partial pressure of oxygen was maintained above 500 mmHg (13). The same group later reinforced these findings in a first-in-man clinical case-study, in which controlled oxygenated rewarming without any oxygen carriers resulted in successful transplantation with good immediate renal function (18). Increasingly, evidence suggests that oxygen carriers may not be required to achieve adequate oxygenation during short-term renal perfusion (17,38,39).

Although beyond the scope of this review that concentrated on normothermic perfusion, there is growing evidence in favor of gradual rewarming. Comparing controlled oxygenated rewarming with continuous up-front perfusion in a porcine transplant model using steen-based solution with 95% oxygen and 5% CO_2_, both methods effectively restored renal function after SCS to the same level, with controlled oxygenated rewarming significantly reducing tenascin C expression in tissue—a glycoprotein induced during injury—compared to SCS (40). Heat-shock proteins are well known as a defense mechanism induced by stressful stimuli such as hypoxia or hyperthemia (41,42). Minor et al. demonstrated that with gradual rewarming (or “controlled hyperthermia”), they found a 50% increase of heat-shock proteins, which correlated to improvement of tubular reabsorption of sodium and glucose upon reperfusion, and reduced loss of urinary protein compared with controls, meriting further exploration of this technique in preclinical models (43). As a result of this work, there is emerging evidence that avoiding the abrupt temperature changes may be protective against IRI.

### Gaseous Composition

Supraphysiological concentrations of oxygen, in the form of 95% O_2_, 5% CO_2_ gas mixtures, have been utilised in most EVNP protocols. However, excess oxygenation may exacerbate IRI through increased production of ROS (4). A porcine kidney transplant model comparing EVNP with 95%, 25% and 12% O_2_ with 5% CO_2_, found that while oxygen extraction was significantly reduced, reducing oxygen levels to normoxia did not significantly influence functional parameters or biomarkers of renal injury during reperfusion (19). This directly contradicts previous studies that advocate hyperoxemia (13,18). Importantly, the latter studies used acellular perfusates, signifying that higher oxygen concentrations may be necessary in the absence of oxygen carriers. In either case, theoretically neither hypoxemia nor hyperoxemia should alter renal vasomotor tone in constant CO_2_ concentrations (44); thus, reducing oxygen tensions would not be expected to influence renal function. Further characterisation of oxidative stress in the context of EVNP may enhance this field of research.

Gases are easily absorbed into the blood, and therefore can be utilised as additives to enhance the protective effects of EVNP. In human-sized porcine kidneys, hydrogen sulfide (H_2_S) infusion after 30 min of EVNP reduced oxygen consumption which was restored rapidly after cessation without any short-term indications of histological or biochemical damage (21). With further corroborating evidence, H_2_S supplementation may offer potential in reducing the extent of oxygenation required, facilitating the use of acellular perfusates or normoxic gas mixtures; further work is required, particularly, to exclude any potential long-term toxicity prior to clinical translation.

Other gases that have been utilised include carbon monoxide (CO), which has shown to significantly reduce IRI in experimental models by promoting vasodilation (20); and argon, which despite suggestion that it may potentially reduce IRI by inhibiting IL-8, did not influence renal function when administered during EVNP of porcine kidneys (22), consistent with EVNP models in porcine lungs (45). These findings may be explained by the longer durations of perfusion permitted in the experimental studies, and that benefits of argon may only be quantifiable after prolonged periods.

### Supplementary Composition

Metabolic and energetic substrates are essential for restoration of normal metabolism. Clinical perfusates have been most commonly supplemented with a nutrient solution with insulin, glucose 5%, sodium bicarbonate 8.4%, multivitamins and extracellular fluid (Ringer’s lactate) to replace urine output (6,7,11). Moreover, blood-based perfusates include anticoagulants to prevent clotting within the perfusate tubing circuitry and to reduce risk of graft thrombosis, and vasodilators to reduce transient vascular constriction upon reperfusion with RBCs (46). Furthermore, liver studies have shown that maintenance of optimal microcirculatory homeostasis using vasodilators is a key factor in EVNP (34). There has been limited research, however, evaluating the impact or need for anticoagulants and vasodilators, particularly in the context of acellular perfusates.

Other supplements in the literature have aimed to further ameliorate IRI. Currently, reduction of inflammatory mediators is achieved through integration of hemadsorption technology (CytoSorb) into the EVNP circuit (25). However, such broad-spectrum hemadsorption may potentially remove important anti-inflammatory mediators. An alternative method proposed to reduce oxidative stress is the utilisation of endogenous HO-1; a heat shock protein that catalyses degradation of haem, exerting cytoprotective effects (42). Naturally, HO-1 decreases during SCS due to reduced protein expression under hypothermia (25,26). However, one study showed that addition of cobalt protoporphyrin (CoPP) during normothermic preservation successfully induces HO-1 within clinically appropriate timeframes (26). Of note, some degree of toxicity, presented as reduced urine output and increased proteinuria, was observed at higher concentrations of CoPP, without further without increases in HO-1. Therefore, optimal HO-1 inducers and concentrations need to be explored further. Vitamin C is known to prevent apoptosis, reduce inflammation and endothelial permeability, in addition to enhancing microcirculation. However, in 6-hour animal EVNP models, no improvements in clinical parameters were observed despite a significant reduction in oxidative stress (23), consistent with negative findings of small clinical studies (47). Finally, EPO supplementation has been speculated to lessen IRI by modulation of apoptotic mediators: caspase-3, interleukin-1ß and HSP70 (24). In porcine kidneys subjected to 2-hour of haem-based EVNP, addition of EPO reduced apoptotic cells in tubular lumens and interstitial areas and facilitated renal tissue remodelling (48). While encouraging, these studies were limited by lack of clinically relevant outcome measures and did not address potential adverse effects.

Of note, no data was found on the use of antibiotics or the specific dosing of the aforementioned additives. Additionally, the administration of therapeutics such as regenerative cell therapies was deemed beyond the scope of this review.

### Duration of Perfusion

Optimising perfusion duration may be a critical step in augmenting the benefits of suitably engineered perfusates. As successfully demonstrated in clinical studies, a short period of EVNP (up to 2-hour) is acceptable following a period of SCS (6,7,11,49). However, continuous normothermic perfusion from time of retrieval may permit complete avoidance of cold ischaemic injury. Recent DCD porcine studies have verified the feasibility and safety of prolonged EVNP with near complete exclusion of SCS using whole-blood perfusates for 10-hour in livers (33,50) and acellular Steen solution for 12-hour in lungs (51). Initial evidence in kidneys showed that continuous, 8-hour EVNP is feasible and safe in good quality beating-heart donor kidney grafts, (52) and in a follow-up study on DCD porcine kidneys, the same group demonstrated that continuous 16-hour EVNP with near complete exclusion of SCS was superior to brief EVNP following SCS (28). Furthermore, sub-normothermic 24-hour preservation using acellular Steen solution has been shown to support low and stable vascular resistance whilst providing adequate histological preservation in DCD porcine kidneys (12). Notably, in this study EVNP beyond 5-hour was not feasible when whole blood alone was used, and when diluted with Steen solution, acidosis, hyperkalaemia and necrosis resulted (12). This study was limited by variable warm ischaemic times, use of inconsistent vasodilators, and lack of post-transplant reperfusion outcome measures; however, it may be of interest to further investigate the effects of different perfusates at varying durations.

Despite this emergent potential, no portable devices are yet available for continuous renal EVNP during transportation, unlike the OrganOx metra device that has shown to continuously preserve donor livers for up to 24-hour (50). Logistical burden of machine failure during transport, health-care costs, and complicated transportation procedures would also require consideration. Therefore, to evaluate outcomes of prolonged EVNP in current clinical context, brief, intermediate and prolonged EVNP following 8 h of SCS were compared in similar DCD porcine models (27). All durations maintained stable hemodynamic parameters, however posttransplant serum creatinine was significantly lower after intermediate and prolonged EVNP compared to the brief EVNP. Noticeably, serum creatinine was higher after 1-hour EVNP compared to SCS alone. This may be explained by several mechanisms: 1) 1-hour is insufficient for repair mechanisms; 2) rapid warming from hypothermia is harmful in short-term, as previously discussed; or 3) discrepancies exist due to different transplant models. Despite the higher tier evidence provided by human clinical studies (7), future studies should assess protein expression during prolonged EVNP to ascertain the specific molecular processes, whilst also exploring the feasibility of portable renal EVNP machines.

Debate remains regarding the recirculation of urine versus replacement of urine losses with colloid solution, particularly in the context of longer perfusion durations. Weissenbacher et al. demonstrated that the recirculation of urine permitted stability over a 24-hour normothermic perfusion period with urine recirculation. The control group (*n* = 3) with fluid replacement as per urine loss were unable to be perfused beyond 4–6 h due to an inability to maintain a physiological pH (53). Subsequent work by the same group has confirmed these findings in a porcine model in which urine circulation aided the maintenance of physiological arterial pressure and acid-base homeostasis (54). Proteomic data also suggests urine recirculation may increase glucose metabolism, which may indicate an increase in metabolic activity, potentially protective against IRI (55).

### Study Strengths and Limitations

Due to the exploratory nature of this review, there lacked clear uniformity in the study designs, objectives, and outcome measures evaluated. Furthermore, high study heterogeneity precluded a meta-analysis. Moreover, a large proportion of the selected studies were experimental, yielding lower strengths of evidence and limiting our use of the recognised Cochrane bias risk assessment tool for randomised controlled trials. However, our efforts in screening a large number of databases, with wide eligibility criteria, provided some safeguard against missing relevant studies. Further identification of potentially relevant studies may have been achieved by expanding the eligibility criteria to include studies of sub-normothermic perfusion methods. The term “EVNP” was used throughout this manuscript, however, we acknowledge that the terms normothermic *ex-vivo* kidney perfusion (NEVKP), sub-normothermic kidney perfusion (SNMP), normothermic machine perfusion (NMP) are also used in the literature. Standardisation and reproducibility of terms is an important part of collaboration with new technologies and techniques; importantly, our search strategy accounted for these additional terms.

### Overall Context and Future Direction

EVNP is a technology used for multiple reasons in the solid organ transplant field. “Optimisation” may represent different factors to different ends. For the purposes of kidney viability assessment, short-term perfusion may provide valuable information. Rapid transplantation places the kidney in a more physiological environment and may make longer perfusion undesirable. Prolonged EVNP clearly has the potential to recondition kidneys and regenerate their injured cells/tissue, not to mention the untapped potential for immunomodulation. Prolonged regeneration and immunomodulation would appear likely to require a more adaptive and physiological environment, perhaps with natural biological homeostats such as a liver in circuit, or with advanced sensors and chemical modulation beyond anything applied in the studies discussed in this review. It will perhaps be the adaptability and sensitivity of the circuit in regulating its perfusate composition, that allows the full potential of this therapy to be realised. There remains room for vast innovation and automation in this field even beyond a device such as Organox which is being taken up rapidly in the liver transplant arena.

## Conclusion

EVNP is an evolving technology which has the opportunity to resuscitate and evaluate kidneys prior to transplantation, and the elucidation of ideal perfusate constituents and perfusion duration remain key in the optimisation of this clinical tool. Our findings suggest that Ringer’s lactate or Steen solution supplemented with nutrient and metabolic substrates provide a suitable environment for preservation at normothermia. Given logistical implications, under current protocols, blood-based perfusates may be suboptimal if synthetic HBOCs or acellular perfusates with carbogen gas mixtures are proven to support adequate oxygenation and enable gradual rewarming where continuous renal EVNP to completely bypass SCS is in development. Particularly given that longer perfusion durations (beyond 6 h) may be harmful with the use of red cell-based perfusates. However, this may relate to the limited homeostasis of established EVNP circuits and will clearly need re-evaluation as the many other biochemical parameters of kidney EVNP are optimised by improved technology. There are also emerging roles for supplementary constituents that reduce metabolism and suppress inflammation which are beyond the scope of this review. *Ex-vivo* modulatory interventions represent a brave new world of therapy in transplantation. Extensive further research is required, however, in appropriate transplant models to ascertain clinical benefits.

It is clear that co-ordinated research aims and better collaboration between the many groups involved in this emerging technology would be beneficial to progress. In conclusion, while current clinical protocols have been feasible, there is increasing evidence that there is potential to better define perfusion composition, in particular with use of non-blood-based perfusates, and prolonged duration, to optimise organo-protective benefits of EVNP.

## Abbreviations

CO, carbon monoxide; DBD, donation after brain death; DCD, donation after circulatory death; DGF, delayed graft function; ECD, extended-criteria donor; EPO, erythropoietin; ESRD, end stage renal disease; EVNP, *ex vivo* normothermic perfusion; H_2_S, hydrogen sulfide; HBOCs, Heam-based oxygen carriers; HMP, hypothermic machine perfusion; IRI, ischaemic reperfusion injury; RBC, red blood cells; ROS, reactive oxygen species; SCS, static cold storage; UW, University of Wisconsin.
