# Supplementary material for: Perfusate Composition and Duration of Ex-Vivo Normothermic Perfusion in Kidney Transplantation: A Systematic Review
Source: Transpl Int. 2022 May 11;35:10236. doi: 10.3389/ti.2022.10236 (PMC9130468; doi:10.3389/ti.2022.10236)
Supplement: Supplementary file 1 [file DataSheet1.pdf]

Supplementary Material

Appendix 1: Study Protocol Version 2 .... **1**

Appendix 2: Search Strategy **8**

Appendix 3: Eligibility Criteria **10**

Appendix 1: Study Protocol Version 2

Version 2: 5th December 2019

Title	
1. Title	Perfusate Composition and Duration of Ex-vivo Normothermic Perfusion in Kidney Transplantation: A Systematic Review

Abstract	
2. Structured Summary	

Introduction	
3. Rational	Kidney transplantation is the gold standard treatment for end stage renal failure[1]. However, continued organ donor shortage requires increased use of marginal donor kidneys, obtained from donation after circulatory death (DCD) and extended criteria donors (ECD)[2], defined as any donor ≥60 years or aged ≥50 years plus two of: a history of hypertension, a raised terminal serum creatinine (>1.5 mg/dL) or death from a cerebrovascular accident[3]. This is associated with increased ischaemic complications, which is reflected in higher rates of delayed graft function. Ultimately, there is increased risk of acute rejection and reduce long-term allograft survival[4-9].<br><br>Traditionally, donor organs have been preserved under hypothermic conditions. This simple, inexpensive and reliable method reduces the metabolic rate of the organ. However, downsides include dependence on refrigeration and inevitable cold ischaemic injury and metabolic tissue damage upon reperfusion with oxygenated blood[10-12]. A novel approach to extend the boundaries of transplantation and utilise kidneys from more marginal donors is ex-vivo normothermic perfusion (EVNP). This involves warming the organ to a normal or near normal body temperature to restore metabolism in an oxygenated red-cell,

	plasma-free perfusate. This technology enables the kidney to be maintained in a stable state allowing close observation and assessment of viability. Additionally, therapies can be added to the isolated organ, ex-vivo, to directly manipulate and improve the condition of the kidney, prior to any systemic exposure to the patient. Early studies have shown that this has the potential to reduce inflammation and oxidative injury, and following resuscitation, improve initial graft function[13-14].<br><br>Various clinical protocols utilising different perfusate compositions, such as the Toronto Protocol in lungs, and Liver transplant models have been established. However, the evidence for specific perfusate compositions remains sparse. Numerous preclinical studies have explored the impact different constituents in perfusion models, however, their implementation into clinical practice remains limited. The aim of this present review was to summarise the reasoning for the different clinical perfusates used, and the optimum composition of perfusates that have proven to render favourable clinical outcomes.
4. Objectives	In the context of EVNP<br>1. what are the roles of perfusate constituent in optimising clinically relevant outcomes?<br>2. What are the effect of different durations of perfusion in optimising clinically relevant outcomes??

**Methods**	
5. Protocol & registration	NA
6. Eligibility Criteria	- **Interventions**: different compositions of perfusate<br>- **Samples**: Kidney donor organs (including lab and animal-based studies).<br>- **Setting**: Hospital/secondary care and lab-based studies<br>- **Type of publications**: Research studies and grey literature<br>- **Publication date**: No limits on publication date

	- **Language & Location**: Publications from any location/country will be accepted where English translations are available.<br>- **Outcomes:** Any<br>- **Study designs**: Any study design (would expect observational/cohort studies rather than RCTs)<br>- **Exclusion criteria**: Data/studies including lungs
7. Information Sources	PubMed; Cochrane Library; Medline; Reference checking; Contacting experts in this field; Data from conferences.
8. Search	**Search Strategy**: Two main reviewers (and a third to resolve any disagreements) will access and select both published and unpublished materials in three stages:<br>1. Limited search of databases to identify relevant keywords contained in the title, abstract and subject descriptors with assistance from a librarian<br>2. Terms and synonyms used by the respective databases will be used in an extensive literature search<br>3. Reference list and bibliographies of articles collected in stage 1 and 2 will be searched.<br>4. Most recently published papers will be assess first and will work backwards from there to identify landmark studies and current/up-to-date knowledge in the field.<br>**Keyworks**: (Ex-vivo Normothermic Perfusion) AND (Perfusate) AND (Kidney)
9. Study Selection	Studies will be selected in adherence to the eligibility criteria using the outline search strategy.
10. Data Collection	Manual data extraction from included studies
11. Data Items	- Qualitative vs. quantitative studies<br>- Type of study (experimental, cohort, case-control etc.)<br>- Outcome suggestions: (time to) Macroscopic appearance of organ; weight pre-/post-perfusion weight of organ; arterial/venous blood gases. For kidneys: total urine output; Inulin/Creatinine clearance;

	Lactate; NGAL; EVNP assessment score; Biochemical composition analysis; ± biopsy/Histological analysis.
12. Risk of Bias in individual studies	Cochrane risk assessment tool if applicable
13. Summary Measures	Summary descriptive table
14. Synthesis of results	Qualitative analysis
15. Risk of bias across studies	NA
16. Additional Analyses	NA

**Results presented as per PRISMA Guidelines**

Results

Flow Diagram in accordance to PRISMA

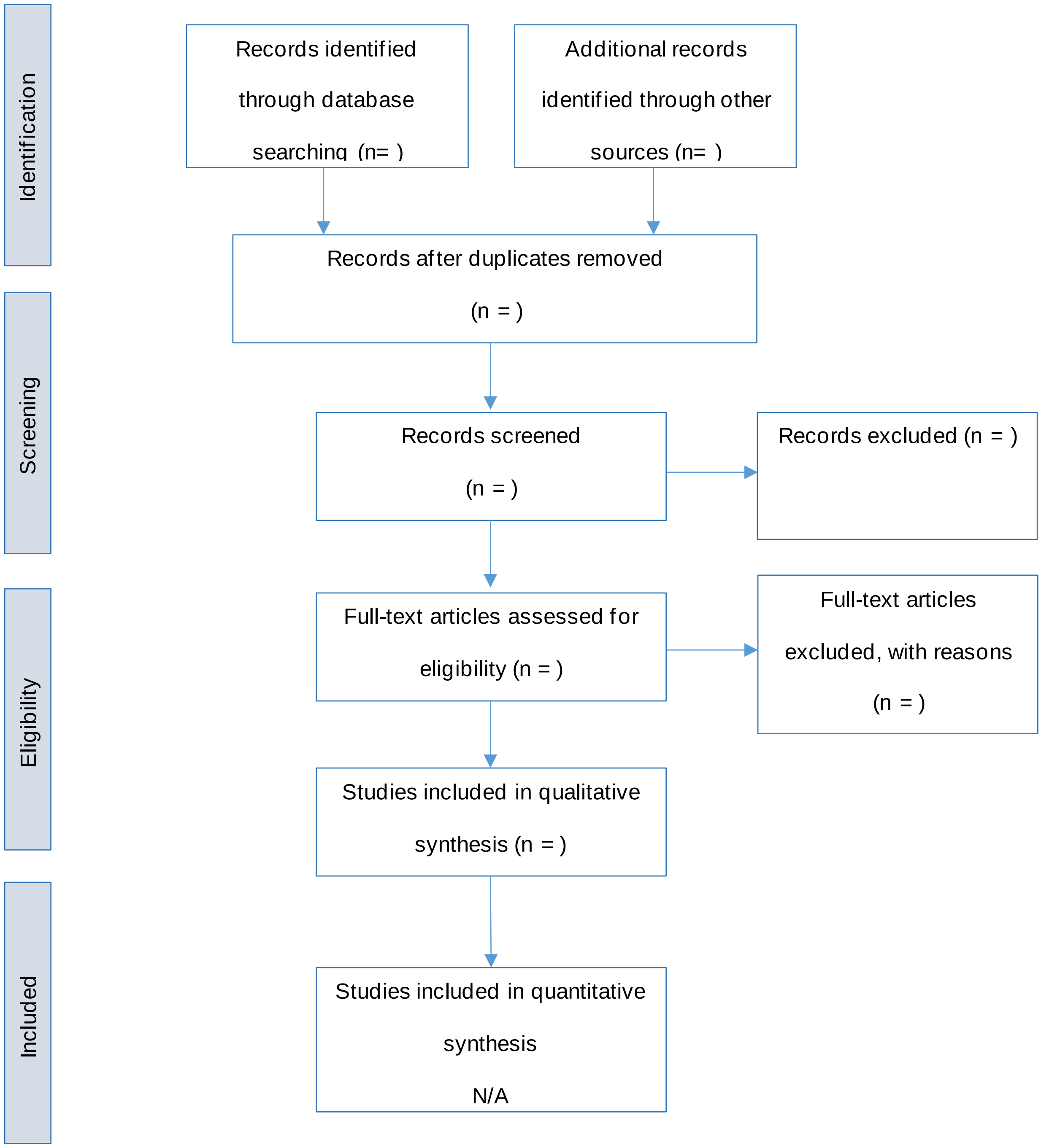

References:

1. Abecassis M, Bartlett S, Collins A, et al. Kidney Transplantation as Primary Therapy for End-Stage Renal Disease: A National Kidney Foundation/Kidney Disease Outcomes Quality Initiative (NKF/KDOQI™) Conference. *Clinical Journal of the American Society of Nephrology*. 2008 Mar; 3(2): 471–480. Available from: doi: 10.2215/CJN.05021107.
2. Perico N1, Ruggenenti P, Scalamogna M, Remuzzi G. Tackling the shortage of donor kidneys: how to use the best that we have. *American Journal of Nephrology. 2003 Jul-Aug;23(4):245-59. Available from: https://www.ncbi.nlm.nih.gov/pubmed/12840600*.
3. Pascual J, Zamora J, Pirsch JD. A systematic review of kidney transplantation from expanded criteria donors. *American Journal of Kidney Disease.* 2008; 52: 553–586. Available from: doi: 10.1053/j.ajkd.2008.06.005.
4. Saidi RF, Elias N, Kawai T, et al. Outcome of kidney transplantation using expanded criteria donors and donation after cardiac death kidneys: Realities and costs. *American Journal of Transplant.* 2007; 7: 2769–2774. Available from: https://www.ncbi.nlm.nih.gov/pubmed/17927805.
5. Van der Vliet JA, Warle MC, Cheung CL, Teerenstra S, Hoitsma AJ. Influence of prolonged cold ischemia in renal transplantation. *Journal of Clinical Transplant and Research*. 2011; 25: E612–E616. Available from: https://doi.org/10.1111/j.1399-0012.2011.01510.x
6. Snoeijs MG, Winkens B, Heemskerk MB, et al. Kidney transplantation from donors after cardiac death: A 25-year experience. *Transplantation* 2010; 90: 1106–1112. Available from: doi: 10.1097/TP.0b013e3181f83b0b.
7. Yarlagadda SG, Coca SG, Formica RN, Jr, Poggio ED, Parikh CR. Association between delayed graft function and allograft and patient survival: A systematic review and meta-analysis. *Nephrology, Dialysis, Transplantation*. 2009; 24: 1039–1047. Available from: doi: 10.1093/ndt/gfn667.
8. Rao PS, Ojo A. The alphabet soup of kidney transplantation: SCD, DCD, ECD–fundamentals for the practicing nephrologist. Clinical Journal of American Society of Nephrology. 2009; 4: 1827–1831. Available from: DOI: https://doi.org/10.2215/CJN.02270409.

9. Quiroga I, McShane P, Koo DD, et al. Major effects of delayed graft function and cold ischaemia time on renal allograft survival. *Nephrology, Dialysis, Transplantation.* 2006; 21: 1689–1696. Available from: https://www.ncbi.nlm.nih.gov/pubmed/16490743.
10. McAnulty JF. Hypothermic organ preservation by static storage methods: Current status and a view to the future. *Cryobiology.* 2010; 60(Suppl): S13–S19. Available from: https://www.ncbi.nlm.nih.gov/pubmed/19538951.
11. Fuller BJ, Lee CY. Hypothermic perfusion preservation: The future of organ preservation revisited? *Cryobiology.* 2007; 54: 129–145. Available from: https://www.ncbi.nlm.nih.gov/pubmed/17362905.
12. Salahudeen AK. Cold ischemic injury of transplanted kidneys: New insights from experimental studies. *American Journal of Physiology. Renal Physiology*. 2004; 287: F181–F187. Available from: https://www.ncbi.nlm.nih.gov/pubmed/15271685.
13. Hosgood SA, Nicholson ML. First in man renal transplantation after ex vivo normothermic perfusion. *Transplantation*. 2011 Oct 15;92(7):735-8. Available from: doi: 10.1097/TP.0b013e31822d4e04.
14. Hosgood SA, Nicholson ML. Renal Transplantation After Ex Vivo Normothermic Perfusion: The First Clinical Study. American Journal of Transplantation. 2013; 13(5). Available from: https://doi.org/10.1111/ajt.12179.

Appendix 2: Search Strategy

1. Source: Embase (Ovid)

   URL: http://ovidsp.ovid.com OR

   http://ovidsp.dc2.ovid.com.ezproxy.lib.gla.ac.uk/sp-

   4.04.0a/ovidweb.cgi?&S=FLEOFPMBFNEBPHPBJPBKBHBHNJOPAA00&Ne

   w+Database=Single%7c4

   Database coverage dates: 1947 to 2021

   Search date: 13/07/2021

   Retrieved records: 1,748

   Search Strategy: (normothermic adj5 perfus*).tw. OR evnp.tw.

2. Source: Ovid Medline® without Revisions

   URL: http://ovidsp.dc2.ovid.com.ezproxy.lib.gla.ac.uk/sp-

   4.04.0a/ovidweb.cgi?&S=FLEOFPMBFNEBPHPBJPBKBHBHNJOPAA00&Ne

   w+Database=Single%7c0

   Database coverage dates: 1996 to 2021

   Search date: 13/07/2021

   Retrieved records: 454

   Search Strategy: (normothermic adj5 perfus*).tw. OR evnp.tw.

3. Source: **Scopus**

   URL: https://www-scopus-

   com.ezproxy.lib.gla.ac.uk/search/form.uri?display=basic

   Database coverage dates: 1996 – 2021

   Search date: 13/07/2021

   Retrieved records: 1,109

   Search Strategy: (normothermic W/5 perfus*) OR (evnp)

4. Source: BIOSIS Previews (Web of Science)

   URL:

   http://apps.webofknowledge.com.ezproxy.lib.gla.ac.uk/BIOSIS_GeneralSearch_input.do?product=BIOSIS&search_mode=GeneralSearch&SID=C3ZuhSNWP154HAUT5cK&preferencesSaved=

   Database coverage dates: 1926 – 2021

   Search date: 13/07/2021

   Retrieved records: 599

   Search Strategy: (normothermic NEAR/5 perfus*) OR (evnp)

Appendix 3: Eligibility Criteria

Inclusion Criteria:

- Published literature;
- Any year;
- Any study designs;
- Any country/region/language where English translations are available;
- Human studies;
- Animal and laboratory studies;
- Perfusate composition subject studies;
- Duration of EVNP;
- Studies of abdominal organs (liver, kidney, pancreas) and the heart screened, however only kidney studies included.

Exclusion Criteria:

- Review articles;
- Non-large mammal studies;
- Lungs, limb and extremity perfusion studies;
- Normothermic regional perfusion studies;
- Extra-corporeal membrane oxygenation studies;
- Perfusate biomarker assessment studies;
- Therapeutic intervention studies. For example, gene therapy, stem cell therapy, nanoparticle therapy, adenoviral vectors, and liver defatting cocktails;
- Temperature of perfusion studies including hypothermic machine perfusion, unless compared to EVNP and meet inclusion criteria.
